# Novel Drug Delivery System Based on Docetaxel-Loaded Nanocapsules as a Therapeutic Strategy Against Breast Cancer Cells

**DOI:** 10.3390/ijms13044906

**Published:** 2012-04-19

**Authors:** Paola Sánchez-Moreno, Houria Boulaiz, Juan Luis Ortega-Vinuesa, José Manuel Peula-García, Antonia Aránega

**Affiliations:** 1 Biocolloid and Fluid Physics Group, Department of Applied Physics, University of Granada, 18071 Granada, Spain; E-Mails: paolasm@ugr.es (P.S.-M.); jlortega@ugr.es (J.L.O.-V.); jmpeula@uma.es (J.M.P.-G.); 2 Human Anatomy and Embryology Department, Regenerative Biomedicine Institute (IBIMER), Campus de la Salud, University of Granada, 18071 Granada, Spain; E-Mail: hboulaiz@ugr.es; 3 Department of Applied Physics II, University of Malaga, 29071 Malaga, Spain

**Keywords:** lipid nanoparticles, docetaxel, drug delivery, breast cancer

## Abstract

In the field of cancer therapy, lipid nanocapsules based on a core-shell structure are promising vehicles for the delivery of hydrophobic drugs such as docetaxel. The main aim of this work was to evaluate whether docetaxel-loaded lipid nanocapsules improved the anti-tumor effect of free docetaxel in breast cancer cells. Three docetaxel-loaded lipid nanocapsules were synthesized by solvent displacement method. Cytotoxic assays were evaluated in breast carcinoma (MCF-7) cells treated by the sulforhodamine B colorimetric method. Cell cycle was studied by flow cytometry and Annexin V-FITC, and apoptosis was evaluated by using propidium iodide assays. The anti-proliferative effect of docetaxel appeared much earlier when the drug was encapsulated in lipid nanoparticles than when it was free. Docetaxel-loaded lipid nanocapsules significantly enhanced the decrease in IC_50_ rate, and the treated cells evidenced apoptosis and a premature progression of the cell cycle from G(1) to G(2)-M phase. The chemotherapeutic effect of free docetaxel on breast cancer cells is improved by its encapsulation in lipid nanocapsules. This approach has the potential to overcome some major limitations of conventional chemotherapy and may be a promising strategy for future applications in breast cancer therapy.

## 1. Introduction

Breast cancer is the most common malignant tumor and the first cause of morbidity and mortality among women worldwide [1,2]. The past century witnessed the maturation of chemotherapy as a viable adjuvant therapeutic modality for the treatment of cancer. Furthermore, in the case of breast disease, there have been advances in its early detection as well as in surgery, radiotherapy, and hormone therapy. The majority of women (>75%) with breast cancer are diagnosed at an early stage or are operable [3], and these patients require adjuvant chemotherapy to reduce the risk of recurrence [4,5].

Taxanes (paclitaxel or docetaxel) are active cytotoxic agents that promote the polymerization of tubulin and the stabilization of microtubules by preventing their disassembly. Adjuvant chemotherapy with taxanes reduces the risk of cancer recurrence and death in patients with early or operable breast cancer and has shown benefits in patients with high-risk node-negative breast cancer. However, although these novel chemotherapeutics have led to improvements in survival, they are associated with numerous drug-related toxicities [6]. Thus, docetaxel has been associated with a significant increase in neutropenia, febrile neutropenia, leucopenia, stomatitis, edema, fatigue and/or asthenia, and diarrhea [6]. These effects are due in part to the high doses used to achieve the desired anti-tumor effect, which are necessary because of the non-specific distribution of both novel and traditional chemotherapies, with only a small fraction of drugs reaching the tumor. It is well known that injected materials suffer from sequestration by the reticuloendothelial system, which is composed of monocytes and macrophages that clear foreign materials [7]. The drugs can accumulate in healthy organs, and there is a fine line between tolerability and severe morbidity, e.g., in the case of doxorubicin, a DNA intercalator that produces cardiotoxicity [8]. Moreover, the commercial formulation of docetaxel, Taxotere^®^, is formulated with a high concentration of Tween 80 (40 g/L), which has been found to have severe side effects, including hypersensitivity reactions, cumulative fluid retention, and nausea and which has shown incompatibility with commonly used polyvinyl chloride intravenous administration sets [9]. Taken together, these factors compromise the curative potential of anticancer drugs, and more effective methods for their delivery to tumors are required [10].

Therefore, novel therapeutic approaches and alternative formulations of docetaxel with no (or low concentration) of Tween 80 are urgently needed to improve the efficacy of treatment in breast cancer patients with a poor prognosis. In this context, liposomes and polymer-drug conjugates were developed in the 1960s and 1970s [11] in attempts to apply Paul Ehrlich’s “magic bullet” concept to chemotherapy [12]. These are now mainstay platforms in nanomedicine, a multidisciplinary field that aims to utilize nanoscale (1–100 nm) constructs to improve the delivery of chemotherapeutics [13]. Primarily, these carriers assist in drug solubilization and protect the drug from degradation. Their nanoscale size range and the frequently ubiquitous presence of poly (ethylene glycol) (PEG) or Pluronic^®^ F68 on their surface aids evasion of the reticuloendothelial system, allowing drugs to accumulate in tumors through enhanced permeability and retention, the result of tumor blood vessel leakiness [14,15]. Lower sequestration rates in healthy organs coupled with higher accumulation and retention rates in tumors would improve the effectiveness of treatments and minimize adverse effects.

Lipid nanocapsules based on a core-shell structure are promising vehicles for the delivery of hydrophobic drugs such as docetaxel. This study is the second part of a broader investigation of lipid nanocapsules. The first part was focused on the physicochemical characterization of three different lipid nanosystems [16]. The main aim of the present study was to evaluate whether docetaxel-loaded lipid nanocapsules improve the anti-tumor effect of free docetaxel in MCF-7 breast cancer cells. Indeed, we found a greater reduction in IC_50_ rate when the docetaxel was encapsulated in lipid nanoparticles, with evidence of apoptosis and premature cell cycle progression from G(1) to G(2)-M phase.

## 2. Results and Discussion

In cancer therapy, most proposed formulations present certain drawbacks related to low drug loading, toxicity, and/or an unsuitable release pattern [17]. Furthermore, as previously noted, docetaxel is formulated using Tween 80 (polysorbate 80) and ethanol (50:50, v/v), and acute hypersensitivity reactions have been noted in the majority of patients treated in phase I clinical trials, [18–20]. Fluid retention and hypersensitivity, among other reactions, are also associated with some other drugs with Tween 80 in their formulation [17]; therefore, this excipient may be partially responsible for some of the toxic effects observed with docetaxel [21,22]. An ideal formulation should provide biocompatible nanosized particles and high drug loading with sustained-release characteristics, allowing drug release in the target site at a therapeutic concentration, thereby minimizing drug inefficiency and adverse effects. Various approaches have been investigated in this line [21,23,24].

In the present study, we reformulated docetaxel in a better-tolerated and less-toxic vehicle to avoid premedication with corticosteroids and antihistamines, which is usually prescribed to reduce adverse reactions associated with docetaxel in Tween 80. Three previously obtained and characterized nanocapsule systems (16) were used to encapsulate docetaxel. All of them have a hydrophobic core constituted by olive oil, but they differ in their shell components. The shell of the simplest system (Epi), with an average diameter of 170 ± 20 nm, comprised a mixture of phospholipid molecules (Epikuron) and a poloxamer (Pluronic^®^ F68), which both act as colloidal stabilizers. Pluronic^®^ F68 possesses the capacity to avoid opsonin adsorption, thereby preventing the triggering of a cascade of immune system reactions that would eliminate the nanocapsules from the bloodstream [15]. Furthermore, because this poloxamer is not toxic, it can be used in nanoemulsions for the release of drugs and genes [25]. A second system (CS), with an average diameter of 330 ± 30 nm, was formulated by replacing the poloxamer with chitosan oligomers. Chitosan is a polysaccharide obtained from the deacetylation of chitin, which is a structural element of the exoskeleton of crustaceans. It is hydrophilic, biocompatible, and biodegradable and has low toxicity, and its effectiveness as an adsorption-enhancing agent has been demonstrated by various authors [26]. Finally, a third formulation (PhS), with an average diameter of 210 ± 20 nm, was designed to create a nanosystem in which Epikuron was replaced with phosphatidyl-serine, producing a carboxyl-functionalized nanosystem that offers the possibility of the efficient attachment of antibody molecules for future vectorization purposes [16]. The surface electrical state of the different nanocapsules was analyzed by measuring their electrophoretic mobility as a function of the pH in low ionic strength media. Electrophoretic mobility is an experimental parameter directly related with the zeta potential and the surface potential, which is dependent of the surface composition and the salinity and pH of the medium. The zeta potential values at neutral pH agree with the nature of the shell of the nanocapsules, showing a positive surface charge (16.3 mV) for CS nanosystem, and a negative surface charge for Epi and PhS nanosystems (−56.4 mV and −44.4 mV respectively).

### 2.1. Uptake Analysis

**Figure 1:**
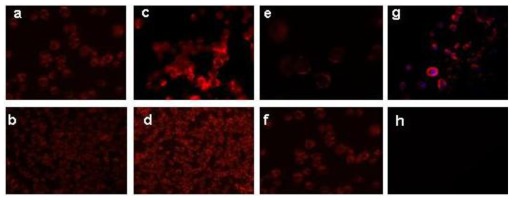
MCF-7 cells treated with: Nile Red- EPI nanocapsules for (**a**) 1 h and (**b**) 3 h; Nile Red-CS nanocapsules for (**c**) 1 h and (**d**) 3 h (f); or Nile Red-Phs nanocapsules for (**e**) 1 h and (**f**) 3 h; (**g**) Localization of Nile Red in the cytoplasm; the cell nucleus is stained with DAPI; (**h**) Blank: cells treated with empty nanocapsules. (**a**, **c**, **e**, **f** and **g**: ×40 magnification; **b**, **d**: ×20 magnification).

Fluorescence microscopy studies were conducted to test the intensity of uptake by MCF-7 cells of the encapsulated Nile red. The uptake was significantly faster for Nile Red-CS nanocapsules than for the other two types. After only 1 h, CS particles showed the highest uptake, followed by Epi and then PhS particles (Figure 1). Uptake increased with longer incubation time, preserving these differences among particles until 5 h, when the cells showed the same amount of fluorescence, and no differences among the three types of nanoparticles could be detected. At 3 h of incubation, the Nile red was localized in the cytoplasm of cells (Figure 1), indicating that these nanosystems are effective vehicles to transport the molecules within MCF-7 cells by endocytosis, and can avoid efflux by cell membrane MDR transport proteins such as p-glycoprotein (P-gp) [25,27,28]. The variation in internalization among the different nanoparticles may be attributable to a combination of surface charge and hydrophilicity, which play a major role in affinity in the endocytosis pathway [29]. The CS nanocapsules showed the highest fluorescence intensity, related to their positive superficial charge, which increases the affinity between nanoparticle and negatively-charged cell membrane [30]. This finding is consistent with the enhanced mucoadhesive properties reported for chitosan nanosystems [31,32]. The lower fluorescence response observed with the EPI and PhS systems appears attributable to the size and negative surface charge of these nanocapsules, as well as to the hydrophilic nature of the poloxamer molecules, which produce systems that are more biocompatible but have a lower cell uptake [33,34]. The lower fluorescence intensity of PhS *versus* EPI nanocapsules may be attributable to the lesser hydrophilicity of the former. Nevertheless, given that these differences disappear after 5 h of treatment, all three systems achieve similar uptake efficacy (data not shown).

### 2.2. Synthesis, Encapsulation Efficiency, and Cytotoxicity Assays of Docetaxel-Loaded Nanoparticles

**Figure 2:**
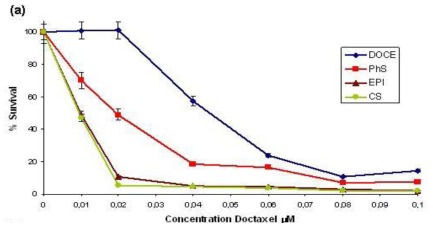

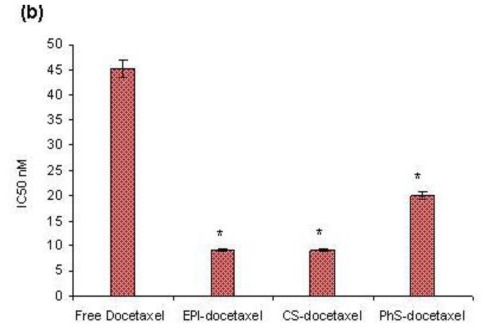
(**a**) Growth-inhibition curves of MCF-7 cells after 6 days of exposure to free docetaxel and to the three docetaxel-loaded nanoparticles (Epi, CS and PhS). Values are the mean of four independent experiments; (**b**) IC_50_ values (drug concentration producing 50% reduction in absorbance in control cells) of free docetaxel and the three docetaxel-loaded nanoparticles, expressed in nM and estimated as described in Material and Methods. * Statistically significant.

Docetaxel-loaded nanoparticles were prepared by using a solvent-displacement technique. For encapsulation, docetaxel was dissolved in olive oil from the organic phase of the emulsion before it was mixed with the aqueous phase. High-performance liquid chromatography (HPLC) study of the encapsulation efficiency of docetaxel showed that the organic phase of CS, Epi, and PhS nanoparticles contained 86%, 80%, and 80% of the drug, respectively. In cytotoxicity assays, MCF-7 cells were treated with free docetaxel or increasing concentrations of the different docetaxel-loaded nanocapsules for 6 days, followed by calculation of the inhibitory concentration 50 (IC_50_) in each case. No significant differences in growth patterns were found between parental MCF-7 cells and MCF-7 cells treated with empty CS, Epi, or PhS nanoparticles (data not shown). With equivalent drug loading in the culture medium, a markedly lower proliferation rate was observed in the MCF-7 cells treated with docetaxel-loaded nanoparticles than in the parental MCF-7 cells treated with free docetaxel (Figure 2). A 20 nM dose of free docetaxel had no cytotoxic effect on cells, whereas 20 nM of docetaxel-loaded CS, Epi, or PhS nanoparticles induced a significant decrease of 94.65%, 89.27%, or 51.01%, respectively, in their survival rate (*P* < 0.001) (Figure 2a). IC_50_ values were 45 nM for free docetaxel, 9 nM for docetaxel-loaded CS and Epi nanoparticles, and 20 nM for docetaxel-loaded PhS nanoparticles (Figure 2b).

Recent studies reported similar effects with high-dose docetaxel in oily core nanocapsules. Youm *et al*. [35] observed that the bioactivity against SUM 225 cells was higher for free *versus* encapsulated docetaxel at lower concentrations (2.5 μM), but was significantly higher for the encapsulated form at the highest concentration (5 μM). Likewise, Li *et al*. [36] found that cell death was greater in drug-resistant MCF-7/ADR cells when doxorubicin was delivered in LA-TPGS nanoparticles at high drug concentrations. Importantly, the Epi, CS, and PhS nanocapsules described in the present study induced high cell death levels even at low concentrations, exerting a five-fold (Epi and CS) and 2.26-fold (PhS) more potent cytotoxic effect *versus* free docetaxel. These data indicate that the sustained release of the drug and its enhanced drug internalization by MCF-7 cells when in this nanocapsule formulation may increase the biological response to docetaxel and allow a decrease in the dose, thereby reducing its side effects.

### 2.3. Apoptosis Determinations and Cell Cycle Analysis

Apoptosis is considered the main cell death mechanism in response to taxanes. Docetaxel targets tubulin, stabilizing microtubules and thereby inducing cell-cycle arrest and apoptosis [37]. We therefore studied these well-documented effects on cell cycle distribution and apoptosis in the tumor cells. When undergoing apoptosis, cells externalize phosphatidylserine, a lipid found on the inner surface of the cell membrane that can be labeled with fluorochrome-conjugated annexin V. It is therefore possible to distinguish between intact cells (stained negative for both annexin V-FITC and propidium iodide [PI]), early apoptosis (stained positive for annexin V-FITC and negative for PI), late apoptosis or cell death (stained positive for both annexin V-FITC and PI), and necrosis (stained positive for PI).

**Figure 3:**
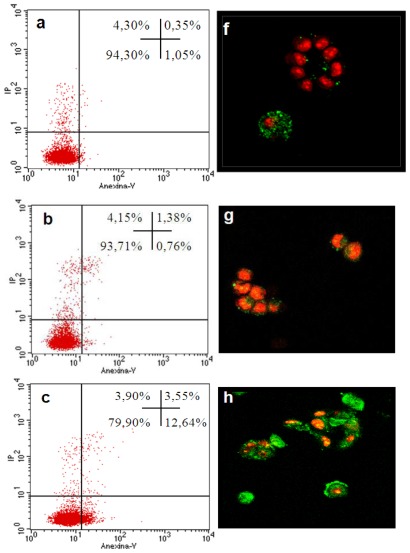

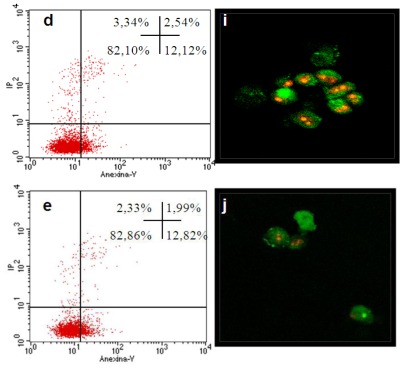
Quantification of *docetaxel-*induced apoptosis in MCF-7 cells by fluorescence-activated cell sorting analysis (**a**, **b**, **c**, **d** and **e**) and confocal laser scanning microscopy (**f**, **g**, **h**, **i**, and **j**). Cells were incubated with free docetaxel or docetaxel-loaded Epi. CS, or PhS nanoparticles for 24 h. Untreated cells served as controls. Cells were stained with annexin V and PI to evaluate apoptotic cell death (see Materials and Methods): (**a** and **f**) MCF-7 control cells, (**b** and **g**) free docetaxel, (**c** and **h**) docetaxel-loaded Epi, (**d** and **i**) docetaxel-loaded CS, (**e** and **j**) docetaxel-loaded PhS. On confocal laser scanning, early apoptosis (stained positive for annexin V-FITC and negative for PI), late apoptosis or cell death (stained positive for both annexin V-FITC and PI) and necrosis (stained positive for PI) can be observed; viable cells are not visible (stained negative for both annexin V-FITC and PI). Data are representative of four separate experiments.

Our flow cytometry study with annexin V assay showed that 94.30% of control MCF-7 culture cells were viable, 1.05% were early apoptotic, 0.35% were in late or final stages of apoptosis, and 4.30% were necrotic (*P* < 0.001) (Figure 3). No significant differences *versus* control cells were found in MCF-7 cells treated for 24 h with empty nanoparticles (data not shown). No apoptosis was detected in cells treated with 45.2 nM free docetaxel for 24 h. Significant apoptosis levels were observed in cells treated with docetaxel-loaded nanoparticles, and the highest apoptosis level (16.19%) was in the cells treated with 9.1 nM of Epi docetaxel-loaded nanoparticles (Figure 3a–e). The confocal microscopy study confirmed these results (Figure 3f–j). In fact, it is known to be difficult to demonstrate programmed cell death by known apoptosis-inducing agents in the MCF-7 human breast cancer cell line, and only a few cytotoxic agents have been found to act preferentially via an apoptotic mechanism in human breast cancer cells [38,39].

**Figure 4:**
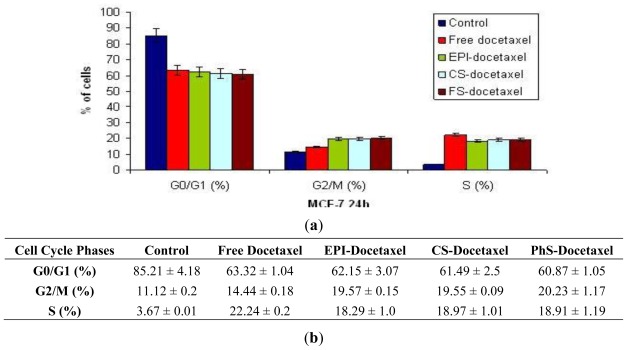
Representative flow cytometry profiles of the cell cycle phase distribution of MCF7 cells (**a,b**). Cells were incubated with free docetaxel or docetaxel-loaded Epi, CS, or PhS nanoparticles for 24 h. Untreated cells served as controls. Cells were subsequently fixed and stained with propidium iodide for DNA content analysis. Data are representative of four separate experiments.

It has also been reported that docetaxel acts at molecular level by impairing mitosis and inducing cell-cycle arrest [40]. We performed flow cytometry studies to determine differences in cell cycle distribution among the treatments. Figure 4 depicts the results of treating MCF-7 cells for 24 h with free or nanocapsule-loaded docetaxel at IC_50_ values. All treatments induced accumulation in G_2_/M and S phases, with a significant decrease in G_0_/G_1_ phase *versus* control cells. The accumulation was significantly greater in cells treated with docetaxel-loaded nanoparticles than in cells treated with the free drug (Figure 4). The rapid decrease in cell cycle progression, evidenced by the increased percentage of cells in S and G_2_/M phase, is in agreement with previous reports that docetaxel treatment induces a G_2_ cell cycle buildup in several cell lines, including MCF-7 [41–43].

## 3. Experimental Section

### 3.1. Cell Lines and Culture Conditions

The human breast cancer MCF-7 cell line was grown at 37 °C in an atmosphere containing 5% CO_2_ with Dulbecco’s modified Eagle Medium (DMEM) (Gibco, Grand Island, NY, USA) supplemented with 10% (v/v) heat-inactivated fetal bovine serum (FBS) (Gibco) 2% l-glutamine, 2.7% sodium bicarbonate, 1% Hepes buffer, and 1% penicillin/streptomycin solution (GPS, Sigma).

### 3.2. Lipid Nanocapsule Preparation

Lipid nanosystems were prepared and characterized as described in [16]. Three systems were formulated: EPI nanocapsules, with surface shell composed of Epikuron and Pluronic^®^ F68; CS nanocapsules, with shell of Epikuron and chitosan oligomers; and PhS nanocapsules, with shell of phosphatidyl-l-serine and Pluronic^®^ F68. For nanosystem characterization, the average size was determined by combining a high-performance-particle-sizer technology with a non-invasive-backscattering method, in which the measurement is taken at an angle of 173°. A Zetasizer-Nano analyzer (Malvern Instruments, Worcestershire, UK) was used to measure the electrophoretic mobility (μ_e_). Zeta potential was calculated from this experimental magnitude in physiological medium conditions.

Fluorescent lipid nanocapsules were formulated by dissolving Nile Red in the olive oil phase at a concentration of 0.025% (w/w). Encapsulation of this fluorescent molecule was confirmed by using a SPEX Fluoromax-2 spectrofluorometer. Emission fluorescence spectra were determined from 550 to 700 nm at an excitation wavelength of 485 nm, with a scanning speed of 100 nm/min and wavelength accuracy of ±1 nm.

Docetaxel-loaded lipid nanocapsules were formulated by dissolving docetaxel in the olive oil phase at a concentration of 0.1% (m/w). The encapsulation efficiency was calculated by HPLC at the Scientific Instrumentation Center of the University of Granada, using a SHIMADZU LC-20AC chromatograph with SPD-M20A diode array detector and C8 Nova-Pak Cartridge column (4 microns, 4.6 × 150 mm); detection was performed at a wavelength of 230 nm.

### 3.3. Uptake Studies

MCF-7 cells (5 × 10^3^) were seeded into 6-well plates under the culture conditions detailed above. After 24 h, cells were fed with fresh medium and treated with Nile Red nanocapsules. Cells were incubated with Nile Red labeled particles for 1 h and 5 h and then washed twice with acidic phosphate saline buffer (PBS) to remove free nanocapsules. Fresh PBS was added and living cells were analyzed by fluorescent microscopy (Nikon Eclipse Ti-S). Cells treated with empty nanoparticles were used as controls.

### 3.4. Cytotoxicity Assays *in Vitro*

MCF-7 cells (1 × 10^3^) were plated into 6-well plates under the culture conditions detailed above. The cells were fed with fresh medium and increased concentrations of free and encapsulated drug every alternate day up to the end of the experiment. After 6 days of treatment, cells were counted using sulforodamine-B (SRB) colorimetric assay as previously described [44] using a Titertek Multiscan apparatus (Flow, Irvine, CA, USA) at 492 nm.

We evaluated the linearity of the SRB assay with cell number using stock MCF-7 cells before each cell growth experiment. IC_50_ values were calculated from semi-logarithmic dose-response curves by linear interpolation. All experiments were plated in triplicate wells and were carried out at least twice.

### 3.5. Apoptosis Detection by Staining with Annexin V-FITC and Propidium Iodide

The Annexin V-FITC Apoptosis Detection kit I (Pharmingen, San Diego, CA, USA) was used to detect apoptosis by flow cytometry. MCF-7 cells (1 × 10^6^ cells) were plated onto 75-cm^2^ flasks and cultured overnight, followed by incubation with IC_50_ values of free or encapsulated docetaxel for 24 h. Cells were harvested by PBS-EDTA, washed twice in cold PBS (1.4 M NaCl, 27 mM KCl, 100 mM KH_2_PO_4_/K_2_HPO_4_, pH 7.2), and pelleted by centrifugation at 500 g for 10 min. They were then resuspended at 10^6^ cells/100 μL in a binding buffer (Hepes buffer, 10 mM, pH 7.4, 150 mM NaCl, 5 mM KCl, 1 mM MgCl_2_, 1.8 mM CaCl_2_), stained with annexin V incubation reagent (1 μL annexin V-FITC (25 μg/mL), 10 μL binding buffer, 10 μL PI (50 μg/mL), and 79 μL H_2_O) and incubated in the dark for 15 min at room temperature. Then, 500 μL binding buffer was added and the cells (10,000 cells per sample) were immediately analyzed using a FACScan flow cytometer (Becton Dickinson, San Jose, CA, USA). All experiments were performed in triplicate and yielded similar results.

### 3.6. Cell Cycle Distribution Analysis

Cells at 70% of confluence were treated with the half maximal inhibitory concentration (IC_50_) of free or encapsulated docetaxel. Cells in monolayer culture were harvested, washed twice with sample buffer (100 mg glucose; 100 mL PBS without Ca^2+^ or Mg^2+^), and fixed in 70% (v/v) cold ethanol for up to 1 week. Cells were pelleted, washed once with sample buffer, and resuspended in PI solution (50 μg/mL PI, 0.5 mg/mL RNase in sample buffer, pH 7.4) for 30 min in the dark. Fluorescence activated cell sorting (FACS) analysis was performed after 24 h of treatment. All experiments were performed in triplicate and yielded similar results.

## 4. Conclusions

In conclusion, our three lipid core-shell nanocapsules appear to be excellent platforms for the encapsulation and delivery of docetaxel into human cancer cells, especially in cancers that are drug-resistant through efflux pump activity. Our carriers, especially the CS and Epi nanoparticles, favor a fast and efficient uptake of the encapsulated drug into tumor cells. Moreover, the encapsulated docetaxel maintained its full activity and preserved its mechanism of action, characterized by apoptosis and premature cell cycle progression from G(1) to G(2)-M phase. Our results suggest that the use of lipid nanocapsules may allow doses of docetaxel to be decreased without loss of therapeutic effect, thereby reducing the drug toxicity. It also avoids any adverse effect of excipients (e.g., Tween 80), and may constitute a promising strategy for future applications in breast cancer therapy.
